# Cost effectiveness of sodium zirconium cyclosilicate for the treatment of hyperkalaemia in patients with CKD in Norway and Sweden

**DOI:** 10.1186/s12882-022-02903-7

**Published:** 2022-08-12

**Authors:** Kun Kim, Josefine Fagerström, Gengshi Chen, Zoya Lagunova, Hans Furuland, Phil McEwan

**Affiliations:** 1 AstraZeneca, Södertälje, Sweden; 2 Karolinska Institutet, Stockholm, Sweden; 3 AstraZeneca, Academy House, 136 Hills Road, Cambridge, CB2 8PA UK; 4 AstraZeneca, Oslo, Norway; 5 Department of Medical Sciences, Uppsala University Hospital, Uppsala, Sweden; 6 HEOR Ltd, Cardiff, UK

**Keywords:** Hyperkalaemia, CKD, Potassium binder, RAASi optimisation, Cost effectiveness

## Abstract

**Background:**

Hyperkalaemia is common in patients with chronic kidney disease (CKD) and is associated with a range of adverse outcomes. Historically, options for management of chronic hyperkalaemia in the outpatient setting have been limited. Novel oral potassium binders provide a safe, effective therapy for maintenance of normokalaemia in patients with CKD, but despite being approved for reimbursement in many countries, prescription data indicate uptake has been slower than anticipated. This analysis aimed to demonstrate the value to patients and the healthcare system of the potassium binder sodium zirconium cyclosilicate (SZC) for treatment of hyperkalaemia in patients with CKD in Norway and Sweden.

**Methods:**

A published simulation model reflecting the natural history of CKD was adapted to the Norwegian and Swedish settings and used to predict long-term health economic outcomes of treating hyperkalaemia with SZC versus usual care.

**Results:**

SZC was highly cost effective compared to usual care in Norway and Sweden, with incremental cost-effectiveness ratios of €14,838/QALY in Norway and €14,352/QALY in Sweden, over a lifetime horizon. The acquisition cost of SZC was largely offset by cost savings associated with reductions in hyperkalaemia events and hospitalisations; a modest overall increase in costs was predominantly attributable to costs associated with gains in life years compared with usual care. SZC remained cost effective in all scenarios examined.

**Conclusions:**

SZC was estimated to be cost effective for treating hyperkalaemia. Consequently, improving access to a clinically effective, safe and cost-effective therapy, such as SZC, may result in considerable benefits for CKD patients with hyperkalaemia.

**Supplementary Information:**

The online version contains supplementary material available at 10.1186/s12882-022-02903-7.

## Background

Hyperkalaemia is an electrolyte abnormality defined as a serum potassium (K^+^) level above the normal physiological range of 3.5–5.0 mmol/L [1, 2]. As renal secretion is the main route of potassium elimination, patients with renal and metabolic comorbidities are at an increased risk of hyperkalaemia [3, 4]. Mild hyperkalaemia is usually asymptomatic, however even modestly raised serum K^+^ (5.0–5.5 mmol/L) is associated with increased risk of adverse outcomes in patients with chronic kidney disease (CKD) [5–7]. This risk profile may partly be compounded by the relationship between renin–angiotensin–aldosterone system inhibitors (RAASi) and hyperkalaemia. International and national guidelines recommend RAASi as first-line agents [8–10] to delay CKD progression and to lower risks of kidney failure, cardiovascular disease and mortality [11, 12]. However, despite these benefits, RAASi – which comprise angiotensin converting enzyme inhibitors (ACEi), angiotensin receptor blockers (ARBs) and mineralocorticoid receptor antagonists (MRAs) – can cause hyperkalaemia [13]. Therefore, RAASi therapy is frequently downtitrated or discontinued in patients who experience hyperkalaemia, contributing to significant discrepancies between guideline recommendations and real-world practice regarding RAASi treatment [13–16]. Failure to achieve guideline-recommended doses of RAASi has been associated with adverse outcomes [8, 13, 17]. Thus, there is an established need for safe and effective treatment of hyperkalaemia, in particular to permit maintenance of RAASi therapy.

Although there is a well-defined treatment pathway for acute, life-threatening hyperkalaemia (K^+^ > 7 mmol/L or with serious ECG changes [18, 19]) in the inpatient setting, [1] management regimes and thresholds for intervention for chronic hyperkalaemia are varied, typically region specific, and may suffer from significant limitations. In general, management of chronic hyperkalaemia comprises a combination of limiting K^+^ intake through dietary modification, preventing K^+^ retention by down-titration or discontinuation of medications such as RAASi, and the use of oral K^+^ binders to facilitate K^+^ excretion via the gastrointestinal route [1]. Until recently, oral K^+^ binders were limited to sodium polystyrene sulfonate (SPS), which is used in both the inpatient and outpatient setting in Sweden, and calcium polystyrene sulfonate (CPS), which is used in Norway, however the efficacies of SPS and CPS are uncertain and they are associated with adverse events (AEs) [2, 20–22].

Novel oral K^+^ binders that safely and effectively manage serum K^+^ may be used to achieve guideline-directed doses of RAASi therapy, [1, 2] which has significant potential to improve long-term outcomes in CKD patients [1]. Sodium zirconium cyclosilicate (SZC) is a novel oral K^+^ binder that has been shown in the ZS-003 and HARMONIZE trials [23, 24] and two open-label studies, ZS-004E and ZS-005 [25, 26] to significantly reduce serum K^+^ in patients with hyperkalaemia and to subsequently maintain normokalaemia. SZC has been recommended by Norwegian Medicines Agency for patients with CKD who develop hyperkalaemia due to RAASi treatment and for patients with heart failure (HF) with serum K^+^ ≥ 6.0 mmol/L, and by Dental and Pharmaceutical Benefits Agency for use in Sweden for adults with CKD stage 3–5, with or without chronic heart failure (HF), when resonium is not suitable; and for adults with chronic HF without comorbid CKD [27–29]. The value of SZC at the population level, in terms of the long-term health and economic burden of hyperkalaemia and suboptimal RAASi therapy in CKD patients, has been limited due to modest treatment uptake, particularly in Sweden. Therefore, this study evaluated the cost effectiveness of SZC versus usual care – a combination of RAASi dose adjustments and intermittent SPS/CPS therapy – for the treatment of hyperkalaemia in patients with CKD in Norway and Sweden.

## Methods

### Model overview

This study utilised a published and validated patient level simulation model that reflects the natural history of CKD and long-term health economic outcomes predicted as a function of serum K^+^ levels and adherence to RAASi therapy [30]. Briefly, modelled patients with CKD progress sequentially through health states representing CKD stages 3a–5 based on an annual RAASi-dependent decline in estimated glomerular filtration rate (eGFR; Table 1) [31]. Patients may experience acute hyperkalaemia, cardiovascular (CV) events, hospitalisation or change in RAASi use, according to CKD stage. Change in RAASi use also depends on serum K^+^, while risks of mortality, CV events and hospitalisation additionally depend on both serum K^+^ and RAASi use [30].

**Table 1:** Model parameters

Characteristic	Mean	Standard error^a^
Age (years)	63.33	0.51
Proportion female	0.37	0.02
eGFR (mL/min/1.73 m^2^)	34.61	0.88
*Annual eGFR decline *[31]		
Annual eGFR decline with RAASi	2.34	0.023
Annual eGFR decline without RAASi	3.52	0.035

*eGFR* Estimated glomerular filtration rate, *RAASi* Renin-angiotensin-aldosterone system inhibitors

^a^ Standard errors used for probabilistic sensitivity analysis; base case uses mean values

The published model [30] was adapted to quantify the clinical and economic consequences of introducing SZC in comparison with usual care in Norway and Sweden, using a lifetime horizon and a cycle length of 1 month. Model outputs were costs, life years (LYs), quality-adjusted life-years (QALYs) and incremental cost-effectiveness ratio (ICER; incremental costs/incremental QALYs). Analysis was performed from the payer perspective, using country-specific discount rates (Norway 4%, Sweden 3%) [32, 33].

### Base case analysis

The base case population comprised CKD stage 3b patients with mean eGFR of 34.6 mL/min/1.73 m^2^ at baseline, as in the HARMONIZE trial (ZS-004; NCT02088073; Table 1) [23]. Our base case analysis utilised a simulated cohort of 30,000 patients, to ensure stable point estimates.

Consistent with the trial design, simulated patients entered the model with hyperkalaemia, defined as serum K^+^ ≥ 5.5 mmol/L in the base case. Relative efficacy of SZC versus usual care utilised arm-specific K^+^ trajectories from HARMONIZE for Days 0–3 and Days 4–28, with longer-term efficacy of SZC (Day 29 +) derived from the ZS-005 (NCT02163499) trial (Table S1, Table S2) [23, 25]. In the base case, these trajectories were derived from the subgroup of patients who had serum K^+^ ≥ 5.5 mmol/L at study baseline. Probability of treatment-related AEs with SZC and usual care were derived from ZS-005 and Nasir et al., respectively (Table S3) [25, 34].

Recurrent hyperkalaemia events were simulated [30, 35] when time-dependent serum K^+^ fluctuations resulted in serum K^+^ ≥ 5.5 mmol/L, based on expert opinion from Norwegian and Swedish clinicians. On experiencing a recurrent hyperkalaemia event, patients in both treatment arms incurred costs depending on hyperkalaemia severity (Table S4). Patients experiencing a recurrent hyperkalaemia event in the modelled SZC arm re-initiated SZC and were modelled using the SZC K^+^ trajectories. Patients in the SZC arm returned to ‘usual care’ K^+^ trajectories after each SZC discontinuation. The efficacy of usual care strategies for patients at risk of hyperkalaemia, such as lifestyle modifications and RAASi dose titration, was considered to be implicitly captured by the placebo arm of the HARMONIZE trial, and was not subject to discontinuation.

The modelled maximum treatment length with SZC per hyperkalaemia episode was 4 months, based on the prescription pattern of patiromer (another novel oral K^+^ binder) since 2016 in the US (Source Healthcare Analytics: Longitudinal patient data set from the US: Source Healthcare Analytics Prescription claims data, unpublished). Modelled SZC dosage used the average dose observed in the ZS-005 trial of 7.2 g (± 2.6) per day [25].

Patients exited the model prior to commencing renal replacement therapy (RRT); there is precedent for this in health economic modelling of CKD therapies, because in many jurisdictions dialysis is not cost effective, and incorporation of dialysis costs in the analysis may deny patients with CKD access to effective therapies [36].

As RAASi therapy is recommended for patients with CKD, all patients were on RAASi therapy at model initiation. RAASi dose adjustments or discontinuation occurred when serum K^+^ ≥ 5.5 mmol/L (Table S5), with 50% patients modelled to return to optimal RAASi dose within 42 (± 4.2) weeks [15, 30].

### Adaptation to Norway and Sweden

A cost was applied to each cycle, including a CKD stage-specific cost, estimated on an annual basis; one-off event costs associated with transient clinical events, applied at the time of the event; and arm-specific treatment costs [37–40]. Cost estimates and sources are presented in Tables S4 and S6. Treatment costs were in accordance with retail prices excluding VAT in Norway and retail prices in Sweden, according to national guidelines; discounts to the list price (e.g. through tendering) were not modelled [32, 33]. All costs were adjusted to 2019 currency units for Norway or Sweden, then converted to Euros (1 EUR = 9.66 NOK and 1 EUR = 10.63 SEK) [41].

Mortality was modelled as all-cause mortality (ACM), adjusted by CKD stage, [42] RAASi use [11] and serum potassium level [11, 15]. Background ACM was applied if it exceeded CKD stage-specific ACM, using 2018 age- and sex-specific life tables for Norway and Sweden [43, 44]. Baseline utility was obtained from the general population in Norway and Sweden, respectively, [45–47] then adjusted to multiplicatively reflect health state by CKD stage and clinical events [45–47]. Disutilities due to treatment-related AEs were applied [23, 25, 34]. Utility weights and sources are presented in Table S7.

### Sensitivity and scenario analyses

Scenarios were evaluated to provide insight into the cost effectiveness associated with plausible alternative clinical practice patterns. Specifically, cost effectiveness of initiating treatment at serum K^+^ ≥ 5.1 mmol/L was explored, as well as patients starting in the model with CKD stage 3a or CKD stage 4.

An additional scenario analysis was conducted that allowed patients to progress to RRT, where potassium-associated clinical events were not modelled but dialysis-related complications were captured as part of the cost of dialysis. Dialysis- and transplant-associated disutilities were applied to the baseline utility.

The likelihood of SZC being cost effective was calculated by a stochastic simulation with 1,000 iterations in probabilistic sensitivity analysis (PSA); variables included in the PSA and their associated distributions are summarised in Table S8. The robustness of the model to assumptions about the input parameters and the impact of uncertainty were examined in deterministic sensitivity analyses (DSA).

## Results

### Base case cost-effectiveness analysis

The results of the base case cost-effectiveness analysis are presented in Table 2. The modelled outcomes indicate that each patient in the usual care arm was associated with total lifetime costs of €82,332 in Norway and €62,735 in Sweden. Treatment with SZC was associated with an increase in the total cost per patient of €3,089 in Norway and €3,070 in Sweden, over a lifetime time horizon. Treatment with SZC was also associated with an increase in both LYs and QALYs, with LY gains of 0.339 and 0.354, and QALY gains of 0.208 and 0.214, in Norway and Sweden, respectively. Consequently, the ICER was €14,838/QALY in Norway and €14,352/QALY in Sweden, indicating that SZC is projected to be highly cost effective for the treatment of chronic hyperkalaemia at an assumed willingness to pay (WTP) threshold of €50,000/QALY for both countries.

**Table 2:** Base case cost effectiveness of SZC versus usual care

	**Norway**			**Sweden**		
	**SZC**	**Usual care**	**Difference**	**SZC**	**Usual care**	**Difference**
Total costs	85,421	82,332	3,089	65,805	62,735	3,070
Treatment	13,767	90	13,677	10,490	44	10,446
Adverse events	847	41	806	471	24	447
Hyperkalaemia	9,270	22,371	-13,101	6,440	15,038	-8,598
CKD	35,257	32,790	2,467	21,214	19,697	1,517
RRT^a^	0	0	0	0	0	0
Arrhythmia	1,448	1,395	53	1,149	1,105	44
CV	5,063	4,996	67	6,524	6,348	176
Hospitalisation	10,594	11,255	-660	16,741	17,753	-1,012
RAASi use change	9,174	9,394	-220	2,775	2,725	50
Total LYs	4.840	4.501	0.339	4.966	4.611	0.354
Total QALYs	2.988	2.780	0.208	2.998	2.784	0.214
QALYs	4.002	3.725	0.276	4.030	3.745	0.286
Health state (CKD)	-0.712	-0.658	-0.054	-0.724	-0.668	-0.057
Adverse events	-0.003	-0.003	0.000	-0.003	-0.003	0.0003
Arrhythmia	-0.001	-0.001	0.000	-0.001	-0.001	0.0000
CV	-0.294	-0.279	-0.014	-0.300	-0.285	-0.0154
Hospitalisation	-0.004	-0.005	0.000	-0.005	-0.005	0.0003
Cost/LY	9,119			8,663		
**Cost/QALY (ICER)**	**14,838**			**14,352**		

Threshold for initiating treatment for initial and recurrent hyperkalaemia events: serum K^+^ ≥ 5.5 mmol/L. All costs presented in Euros

^a^ In the base case, all patients exited the model before commencing RRT

*CKD* Chronic kidney disease, *CV* Cardiovascular, *ICER* Incremental cost-effectiveness ratio, *LY* Life year, *QALY* Quality-adjusted life year, *RAASi* Renin–angiotensin–aldosterone system inhibitor, *RRT* Renal replacement therapy, *SZC* sodium zirconium cyclosilicate

The increased treatment acquisition costs of SZC were largely offset by cost savings associated with reduction in hyperkalaemia events and hospitalisations (Table 2). The increased cost associated with SZC overall was largely driven by higher costs associated with CKD in the SZC arm, with small contributions from costs associated with arrythmia, CV events and AEs. The increase in these costs is due to the increased survival in the SZC arm, meaning that patients had more time to experience events and accumulate health state- and event-associated costs. This increased longevity associated with the SZC arm also explains the larger total disutility associated with CKD and CV events in the SZC arm (Table 2).

### Cost effectiveness of alternative clinical scenarios

Given the lack of consensus in clinical practice about the appropriate threshold for initiating treatment, a specific scenario was constructed to evaluate the cost effectiveness of initiating treatment with SZC at a serum K^+^ threshold ≥ 5.1 mmol/L (Table 3). The modelled threshold for both initial and recurrent hyperkalaemia events was defined as serum K^+^ ≥ 5.1 mmol/L and the arm-specific serum K^+^ trajectories were derived from the full HARMONIZE, ZS-004E and ZS-005 trial cohorts [23, 25, 26]. In this scenario, key model drivers were broadly similar to those seen in the base case, however, SZC was associated with both increased incremental costs and smaller QALY gains compared to usual care at a serum K^+^ threshold of ≥ 5.1 mmol/L, compared to the base case threshold of ≥ 5.5 mmol/L. Nevertheless, SZC remained cost effective, with an ICER of €48,862/QALY for Norway and €37,253/QALY for Sweden.

**Table 3:** Cost effectiveness of clinical scenario analyses

Scenario	Model outcomes	Norway			Sweden		
		**SZC**	**Usual care**	**Difference**	**SZC**	**Usual care**	**Difference**
Serum K^+^ treatment threshold ≥ 5.1 mmol/L	Total costs	79,951	73,864	6,087	61,988	57,210	4,777
	Total LYs	4.925	4.728	0.197	5.056	4.849	0.206
	Total QALYs	3.041	2.916	0.125	3.053	2.925	0.128
	Cost/LY	30,958			23,168		
	**Cost/QALY (ICER)**	**48,862**			**37,253**		
Patients initiate with CKD stage 3a	Total costs	104,546	101,983	2,563	82,625	78,926	3,699
	Total LYs	7.207	6.821	0.385	7.500	7.085	0.415
	Total QALYs	4.629	4.384	0.245	4.720	4.461	0.259
	Cost/LY	6,650			8,910		
	**Cost/QALY (ICER)**	**10,457**			**14,259**		
Patients initiate with CKD stage 4	Total costs	57,045	55,733	1,312	42,823	41,634	1,190
	Total LYs	2.690	2.496	0.195	2.720	2.521	0.199
	Total QALYs	1.493	1.383	0.110	1.469	1.359	0.110
	Cost/LY	6,744			5,979		
	**Cost/QALY (ICER)**	**11,908**			**10,798**		
Patients permitted to progress to RRT	Total costs	138,549	128,573	9,976	114,940	105,370	9,570
	Total LYs	7.195	6.558	0.636	7.649	6.954	0.694
	Total QALYs	4.152	3.798	0.354	4.322	3.942	0.380
	Cost/LY	15,679			13,783		
	**Cost/QALY (ICER)**	**28,211**			**25,179**		

All costs presented in Euros. Disaggregated results can be found in the data supplement, Tables S9–S11

*CKD* Chronic kidney disease, *ICER* Incremental cost-effectiveness ratio, *LY* Life year, *QALY* Quality-adjusted life year, *RRT* Renal replacement therapy, *SZC* sodium zirconium cyclosilicate

In the modelled base case, patients entered in the model with CKD stage 3b. Scenarios with patients initiating with CKD stage 3a or 4 were explored (Table 3). In both scenarios, treatment with SZC remained highly cost effective over a lifetime horizon; for CKD stage 3a, the ICERs were €10,457/QALY and €14,259/QALY in Norway and Sweden, respectively, while for CKD stage 4 the respective ICERs were €11,908/QALY and €10,798/QALY.

Patients in the base case exited the model before moving to RRT. In a scenario where patients were permitted to remain in the model and transition from CKD stage 5 to RRT, ICERs remained cost effective in both countries; €28,211/QALY for Norway and €25,179/QALY for Sweden (Table 3).

### Sensitivity analyses

**Fig. 1:**
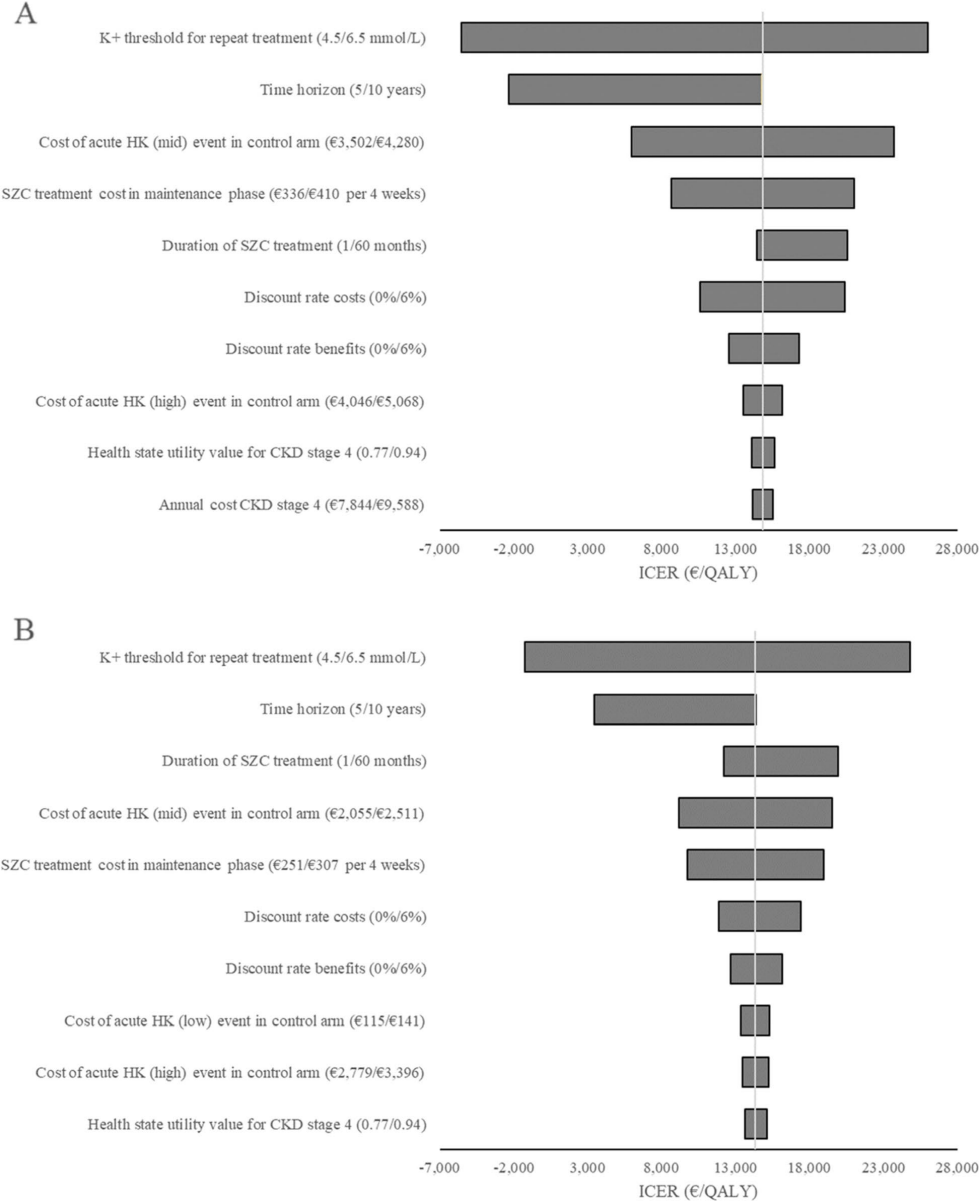
Deterministic sensitivity analysis. **A** Deterministic sensitivity analysis for Norway. **B** Deterministic sensitivity analysis for Sweden. The ten most influential parameters were varied between the upper and lower limits specified in brackets. Negative ICERs represent dominant results i.e. cost savings combined with QALY gains. CKD: chronic kidney disease; HK: hyperkalaemia; ICER: incremental cost-effectiveness ratio; QALY: quality-adjusted life year; SZC: sodium zirconium cyclosilicate

To examine the impact of the assumptions for model inputs on the cost effectiveness, deterministic sensitivity analysis was performed, where individual parameters were varied between lower and upper bounds of the mean values used in the base case. The impact of the ten most influential parameters is illustrated in Fig. 1A (Norway) and Fig. 1B (Sweden). Consistent with the scenario analysis described above, the most influential parameter was the serum K^+^ threshold. When this was varied between 4.5 and 6.5 mmol/L, the ICER ranged from -€5,567 to €26,019 in Norway and from -€1,238 to €24,829 in Sweden (negative numbers indicate dominant results i.e. associated with both QALY gains and cost savings). All scenarios remained cost effective over the ranges explored, implying that the uncertainty in these parameters has little influence on the cost-effectiveness conclusions.

**Fig. 2:**
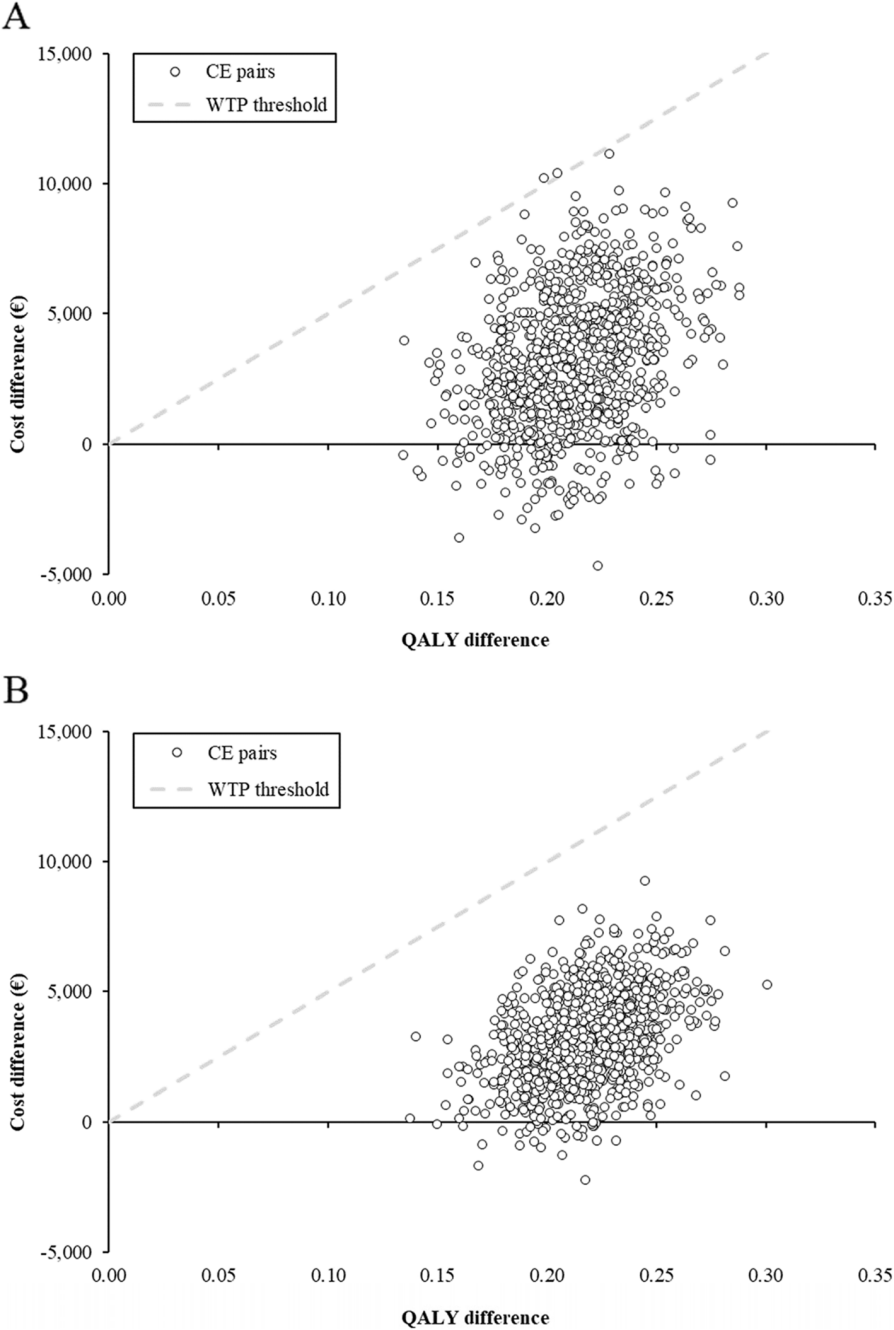
Cost-effectiveness plane for the probabilistic sensitivity analysis. **A** Cost-effectiveness plane for the PSA for Norway. **B** Cost-effectiveness plane for the PSA for Sweden. Each point represents the ICER generated from one of 1,000 model iterations, which were run with the parameters randomly sampled from a distribution around the base case point estimate. Dashed line corresponds to a WTP threshold of €50,000/QALY. CE: cost-effectiveness; ICER: incremental cost-effectiveness ratio; PSA: probabilistic sensitivity analysis; QALY: quality-adjusted life year; WTP: willingness to pay

In PSA, the probability of SZC being cost effective compared to usual care was 99.8% in Norway and 100% in Sweden (Fig. 2).

## Discussion

This study modelled the clinical and economic impact of SZC versus usual care for the treatment of chronic hyperkalaemia in patients with CKD, to evaluate the cost effectiveness of introducing SZC treatment in Norway and Sweden. This analysis adapted an established simulation model [30] that incorporates the natural history of CKD, serum K^+^ fluctuations and changes in RAASi use to inform long-term health economic outcomes. The methods followed recommendations for health economic assessments to provide information for value-based decision making for healthcare services, and demonstrated that SZC is a cost-effective treatment for hyperkalaemia in both Norway and Sweden, compared to usual care, at a range of clinically plausible serum K^+^ treatment thresholds.

Usual care for chronic hyperkalaemia comprises implementation of a low-potassium diet, down-titration or cessation of RAASi and intermittent SPS/CPS, but each of these has significant limitations. Recent KDIGO guidance on hyperkalaemia management in kidney disease highlighted that evidence supporting low-potassium diet is weak and that it may deprive patients of the benefits of a plant-rich diet [1]. Similarly, downtitration or discontinuation of RAASi deprives patients of the benefits of RAAS inhibition and is associated with an increased risk of adverse outcomes [8, 13, 17]. The evidence for the efficacy of SPS/CPS is modest; one small, short-term, single-blinded RCT has been published comparing SPS to CPS, [34] however no comparison with placebo was performed and no studies have evaluated longer term efficacy or safety. Furthermore, SPS has been associated with serious AEs [1] as well as poor palatability; consequently, ESC guidelines recommend not to use SPS in the medium or long term due to the risk of severe gastrointestinal side effects [2, 20–22]. In contrast, SZC has been demonstrated in several trials to effectively treat hyperkalaemia and maintain normokalaemia, including in patients with CKD and those receiving dialysis, and has a favourable safety and tolerability profile [23, 25, 26, 48]. Furthermore, the benefit of using novel oral K^+^ binders to optimise RAASi use has been recognised by the ESC, [2, 8] and this approach may also be beneficial for patients with CKD, as reflected in the 2020 UK Renal Association hyperkalaemia guidelines [49].

In particular, therapies such as RAASi that slow the progression of CKD delay the need to initiate RRT. RRT, particularly dialysis, is associated with reduced quality of life, [50] therefore strategies that delay RRT onset can provide substantial clinical benefit to patients with CKD. In addition to RAASi, other interventions aimed at slowing the progression of CKD are being introduced, such as the SGLT2 inhibitors. Although these have demonstrated efficacy in slowing eGFR decline, [51] it is not anticipated that their introduction will impact on the present analysis. While slowing CKD progression is expected to reduce the overall incidence of hyperkalaemia caused by the increased risk of developing hyperkalaemia as renal function declines, the population considered in the present analysis are those who have already experienced a hyperkalaemia event and are thus at risk of recurrence.

The main limitations of this study relate to the necessary modelling assumptions required due to a lack of published evidence. In particular, as no trials have been conducted directly comparing the efficacy of SZC with either SPS or CPS, nor of SPS or CPS with placebo, we assumed the placebo arm of the HARMONIZE trial was representative of usual care during the maintenance phase. Intermittent SPS treatment is used on an outpatient basis in Sweden; it is unlikely that the data modelled fully capture this, therefore the efficacy may be underestimated in the usual care arm for the Swedish setting. Furthermore, a low-potassium diet was not mandated in the SZC trials, therefore it is uncertain to what extent the potential efficacy of such a diet may impact either the modelled SZC or usual care arms. However, as evidence supporting the efficacy of low-potassium diets is weak and adherence is challenging, our approach may be considered reflective of real-world practice. Additionally, patients in the SZC trials received treatment for concomitant acidosis as required, therefore the effect of this on hyperkalaemia is also implicitly captured in the modelling.

Where there was uncertainty in parameters, the base case sought to be conservative. For instance, the K^+^ trajectories of usual care from Day 29 + were assumed to be equal to Days 15–28, although it is likely that K^+^ levels would elevate as CKD progresses. Additionally, no evidence was found to allow a specific disutility of hyperkalaemia to be modelled, even though hyperkalaemia is associated with adverse outcomes [5–7]. Another conservative assumption was that CKD patients with comorbid HF do not derive additional benefit on HF disease progression from maintaining RAASi.

An additional limitation is that this analysis did not consider patiromer, another novel oral K^+^ binder. There is no head to head comparison between SZC and patiromer; while similar efficacy and safety profiles for normokalaemia maintenance are reported, [25, 52] patiromer’s slower onset of action means it may not be suitable for rapid control of hyperkalaemia [53]. There are additional benefits to patients and healthcare providers that were not captured in the cost-effectiveness analysis: SZC, unlike patiromer, can be stored at room temperature, and has a less restricted dosing window, which are meaningful in terms of facilitating the logistics of dispensing services and reducing the medication burden for patients with CKD. Additionally, SZC is licensed for use in patients receiving dialysis, and is particularly relevant for those dialysis patients who become anuric.

The HARMONIZE trial population was younger and contained a higher proportion of males compared to data published on a real-world Danish cohort of newly diagnosed CKD patients [54]. This is a typical limitation of using clinical trial data. Furthermore, the HARMONIZE trial contained a higher proportion of patients with diabetes than the Danish cohort, [23, 54] however, the trial is still expected to be broadly generalisable to patients and clinical practice in Sweden and Norway, because patients in clinical practice are expected to have a longer history of CKD compared to those enrolled in the Danish study, and the number of comorbidities, including diabetes, and the risk of hyperkalaemia, are expected to increase with longer history of CKD [55, 56].

Therefore, despite these limitations, we consider the analysis to be broadly generalisable to Swedish and Norwegian patients and clinical practice. Another strength was that no extrapolation of efficacy was required; all efficacy data derived from the trial follow-up period. Furthermore, SZC remained cost effective in all clinically-plausible scenarios examined, and the results of the sensitivity analyses indicated that the cost-effectiveness results were robust to assumptions. The highest impact parameter was the threshold of serum K^+^ for initiating active treatment. This was explicitly explored in a scenario analysis where the serum K^+^ threshold for treatment was changed to ≥ 5.1 mmol/L and the K^+^ trajectories were derived from the full HARMONIZE, ZS-004E and ZS-005 data sets [23, 25, 26]. Current clinical practice in Sweden and Norway is to initiate active treatment at a serum K^+^ threshold of 5.5 mmol/L, as in the base case. This choice of threshold represents a clinician-led risk–benefit analysis of mild hyperkalaemia against the AEs, tolerability and poor palatability of SPS/CPS. However, SZC provides an effective alternative with a proven safety and tolerability profile, which may lead to a re-evaluation of the treatment threshold, set against the increase in risks of adverse outcomes associated with serum K^+^ ≥ 5.1 mmol/L. The demonstration of the cost effectiveness of SZC at the lower threshold provides further evidence in support of this.

## Conclusion

Despite being a clinically effective treatment for hyperkalaemia, prescription data, particularly from Sweden, shows that uptake of SZC remains lower than anticipated, suggesting that the potential impact of novel K^+^ binders on clinical care of patients with CKD and hyperkalaemia has yet to be realised. The evidence presented suggests that the acquisition cost of SZC was largely offset by cost savings associated with reductions in hyperkalaemia events and hospitalisations; a modest overall increase in costs was predominantly attributable to costs associated with gains in life years compared with usual care. Overall, SZC was estimated to be cost effective for treating hyperkalaemia with a serum K^+^ threshold ≥ 5.5 mmol/L or ≥ 5.1 mmol/L. Consequently, improving access to a clinically effective, safe and cost-effective therapy, such as SZC, may result in considerable benefits for advanced CKD patients with hyperkalaemia.

## Supplementary Information

**Additional file 1: Table S1. **Mixed effects model parameters to characterise base case serum K^+^ trajectory in SZC and usual care arms. **Table S2.** Mixed effects model parameters to characterise scenario serum K^+^ trajectory in SZC and usual care arms. **Table S3. **Annual probabilities of treatment-related adverse events. **Table S4.** Unit costs for health state by CKD stage and clinical events. **Table S5.** RAASi discontinuation and down-titration. **Table S6.** Treatment related adverse event costs per event. **Table S7. **Health state utilities and events disutilities per cycle (28 days). **Table S8.** Variables and distributions sampled in the PSA. **Table S9.** Disaggregated results of cost effectiveness of scenario with serum potassium treatment threshold ≥5.1 mmol/L. **Table S10.** Disaggregated results of cost effectiveness of scenario with patients initiating in the model at CKD stage 3a or stage 4. **Table S11.** Disaggregated results of cost effectiveness of scenario with patients permitted to progress to renal replacement therapy.

## Data Availability

Data underlying the findings described in this manuscript may be obtained in accordance with AstraZeneca’s data sharing policy described at https://astrazenecagroup-dt.pharmacm.com/DT/Home; contact the corresponding author if further clarification is required. The data analysed during the current study were sourced from published studies and other publicly-available information and are also available in the original publications referenced. The population and efficacy data used were sourced from the HARMONIZE (ZS-004; ClinicalTrials.gov identifier NCT02088073), ZS-004E (ClinicalTrials.gov identifier NCT02107092) and ZS-005 (ClinicalTrials.gov identifier NCT02163499) trials, while adverse event, cost and utility data and their respective sources are provided in the supplementary file.

## Abbreviations

ACEi: Angiotensin converting enzyme inhibitors
AEs: Adverse events
BMI: Body mass index
CKD: Chronic kidney disease
CPD: Chronic pulmonary disease
CPS: Calcium polystyrene sulfonate
CV: Cardiovascular
DSA: Deterministic sensitivity analyses
eGFR: Estimated glomerular filtration rate
HF: Heart failure
ICER: Incremental cost-effectiveness ratio
K^+^: Potassium
LYs: Life years
MACE: Major adverse cardiovascular events
MRAs: Mineralocorticoid receptor antagonists
PSA: Probabilistic sensitivity analysis
PVD: Peripheral vascular disease
QALYs: Quality-adjusted life-years
RAASi: Renin–angiotensin–aldosterone system inhibitors
RRT: Renal replacement therapy
SPS: Sodium polystyrene sulfonate
SZC: Sodium zirconium cyclosilicate
