# Supplementary material for: Cost effectiveness of sodium zirconium cyclosilicate for the treatment of hyperkalaemia in patients with CKD in Norway and Sweden
Source: BMC Nephrol. 2022 Aug 12;23:281. doi: 10.1186/s12882-022-02903-7 (PMC9373488; doi:10.1186/s12882-022-02903-7)
Supplement: Supplementary file 1 — Additional file 1: Table S1. Mixed effects model parameters to characterise base case serum K+ trajectory in SZC and usual care arms. Table S2. Mixed effects model parameters to characterise scenario serum K+ trajectory in SZC and usual care arms. Table S3. Annual probabilities of treatment-related adverse events. Table S4. Unit costs for health state by CKD stage and clinical events. Table S5. RAASi discontinuation and down-titration. Table S6. Treatment related adverse event costs per event. Table S7. Health state utilities and events disutilities per cycle (28 days). Table S8. Variables and distributions sampled in the PSA. Table S9. Disaggregated results of cost effectiveness of scenario with serum potassium treatment threshold ≥5.1 mmol/L. Table S10. Disaggregated results of cost effectiveness of scenario with patients initiating in the model at CKD stage 3a or stage 4. Table S11. Disaggregated results of cost effectiveness of scenario with patients permitted to progress to renal replacement therapy. [file 12882_2022_2903_MOESM1_ESM.docx]

Cost effectiveness of sodium zirconium cyclosilicate for the treatment of hyperkalaemia in patients with CKD in Norway and Sweden

Kun Kim, Josefine Fagerström, Gengshi Chen, Zoya Lagunova, Hans Furuland, Phil McEwan

# Supplementary methods

## Serum potassium trajectories

Parameters used to determine the arm-specific serum K⁺ trajectories in the base case (serum K⁺ threshold ≥5.5 mmol/L) are presented in Table S1, and those for the arm-specific serum K⁺ trajectories in the scenario in which the serum K⁺ threshold was ≥5.1 mmol/L are presented in Table S2.

Table S1. Mixed effects model parameters to characterise base case serum K⁺ trajectory in SZC and usual care arms

| Parameters | Day 0-3 | Day 4-14 | Day 15-28 | | Day 29+ | |
| --- | --- | --- | --- | --- | --- | --- |
| Treatment: SZC | | | | | | |
| Fixed: Intercept | 5.965 | 4.808 | 4.808 | | 4.692 | |
| Fixed: Time (days) | -0.435 |  |  | |  | |
| Random: Patient | 0.275 | 0.340 | | | | |
| Random: Observation | 0.336 | 0.412 | | | | |
| Comparator: Usual care | | | | | | |
| Fixed: Intercept | 5.965 | 5.084 | | 5.355 | | 5.355 |
| Fixed: Time (days) | -0.435 |  | |  | |  |
| Random: Patient | 0.275 | 0.300 | | | | |
| Random: Observation | 0.336 | 0.410 | | | | |
| Base case serum K^+^ trajectory uses a hyperkalaemia threshold of ≥5.5 mmol/L and is derived from data from the subset of patients in the HARMONIZE and ZS-005 trials who had serum K⁺ ≥5.5 mmol/L at study baseline.^1,2^  SZC: sodium zirconium cyclosilicate | | | | | | |

Table S2. Mixed effects model parameters to characterise scenario serum K⁺ trajectory in SZC and usual care arms

| Parameters | Day 0-3 | Day 4-14 | Day 15-28 | | Day 29+ | |
| --- | --- | --- | --- | --- | --- | --- |
| Treatment: SZC | | | | | | |
| Fixed: Intercept | 5.714 | 4.771 | 4.771 | | 4.710 | |
| Fixed: Time (days) | -0.377 |  |  | |  | |
| Random: Patient | 0.322 | 0.339 | | | | |
| Random: Observation | 0.326 | 0.424 | | | | |
| Comparator: Usual care | | | | | | |
| Fixed: Intercept | 5.714 | 4.972 | | 5.194 | | 5.194 |
| Fixed: Time (days) | -0.377 |  | |  | |  |
| Random: Patient | 0.322 | 0.325 | | | | |
| Random: Observation | 0.326 | 0.387 | | | | |
| Scenario serum K^+^ trajectory uses a hyperkalaemia threshold of ≥5.1 mmol/L and is derived from data from the full patient population in the HARMONIZE, ZS-004E and ZS-005 trials.^1-3^  SZC: sodium zirconium cyclosilicate | | | | | | |

## Adverse event probabilities

The probability of treatment-related AEs associated with SZC and usual care are presented in Table S3.

Table S3. Annual probabilities of treatment-related adverse events

| **Adverse event** | **SZC** | | **Usual care** | |
| --- | --- | --- | --- | --- |
|  | **Mean** | **SE** | **Mean** | **SE** |
| Oedema (generalised and peripheral) | 0.116 | 0.012 | 0.060 | 0.034 |
| Worsening hypertension | 0.109 | 0.011 | 0.000 | 0.000 |
| Constipation | 0.064 | 0.009 | 0.120 | 0.046 |
| Diarrhoea | 0.044 | 0.007 | 0.020 | 0.020 |
| Nausea | 0.075 | 0.010 | 0.180 | 0.054 |
| Hypomagnesaemia | 0.012 | 0.004 | 0.000 | 0.000 |
| Anorexia | 0.000 | 0.000 | 0.140 | 0.049 |
| Hypokalaemia | 0.015 | 0.004 | 0.000 | 0.000 |
| Anaemia | 0.059 | 0.009 | 0.000 | 0.000 |
| Urinary tract infection | 0.079 | 0.010 | 0.000 | 0.000 |
| SZC arm probabilities derived from ZS-005 trial.^2^ Usual care arm probabilities derived from Nasir et al (2014).^4^  SE: standard error; SZC: sodium zirconium cyclosilicate. | | | | |

## Healthcare resource use and cost

Costs associated with healthcare resource use are presented in Table S4.

Table S4. Unit costs for health state by CKD stage and clinical events

| **Parameter** | **Norway** | | | **Sweden** | | |
| --- | --- | --- | --- | --- | --- | --- |
|  | **Mean** | **SE** | **Source** | **Mean** | **SE** | **Source** |
| Annual cost: CKD 3a | 4,206.14 | 1,286.99 | ^5^ | 2,898.78 | 886.97 | ^5^ |
| Annual cost: CKD 3b | 4,206.14 | 1,286.99 | ^5^ | 2,898.78 | 886.97 | ^5^ |
| Annual cost: CKD 4 | 8,716.03 | 1,300.14 | ^5^ | 4,890.74 | 729.54 | ^5^ |
| Annual cost: CKD 5 (pre-RRT) | 8,716.03 | 1,300.14 | ^5^ | 4,890.74 | 729.54 | ^5^ |
| Annual cost: Dialysis | 57,540.31 | 5,754.03 | ^5^ | 50,915.47 | 5,091.55 | ^5,6^ |
| One-off cost: Transplant | 51,197.91 | 5,119.79 | ^7^ | 31,798.96 | 3,179.90 | ^6^ |
| Annual cost: Transplant maintenance ^a^ | 646.90 | 64.69 | ^7^ | 513.66 | 51.37 | ^6^ |
| Event cost: Acute hyperkalemia ^b^ (potassium <6 mmol/L) | 74.07 | 7.41 | ^7,8, expert opinion^ | 128.19 | 12.82 | ^9, expert opinion^ |
| Event cost: Acute hyperkalemia ^c^ (potassium between 6.0-6.5 mmol/L) | 3,890.90 | 389.09 | ^7,8, expert opinion^ | 2,282.84 | 228.28 | ^9, expert opinion^ |
| Event cost: Acute hyperkalemia ^d^ (potassium > 6.5 mmol/L) | 4,606.55 | 460.65 | ^7,8, expert opinion^ | 3,087.23 | 308.72 | ^9, expert opinion^ |
| Event cost: Arrhythmia | 3,176.77 | 317.68 | ^7^ | 2,457.46 | 245.75 | ^10^ |
| Event cost: Initial CV event | 4,331.95 | 433.20 | ^7^ | 4,117.13 | 411.71 | ^10^ |
| Annual cost: Post-CV event | 388.53 | 38.85 | ^11^ | 1,206.35 | 120.64 | ^12^ |
| Event cost: Hospitalisation | 2,511.60 | 251.16 | ^5^ | 3,841.12 | 384.11 | ^10^ |
| Event cost: RAASi discontinuation ^e^ | 3,230.05 | 323.00 | ^7,8, expert opinion^ | 870.03 | 87.00 | ^9, expert opinion^ |
| Event cost: RAASi downtitration ^f^ | 2,677.69 | 267.77 | ^7,8, expert opinion^ | 975.51 | 97.55 | ^9, expert opinion^ |
| Event cost: RAASi uptitration ^g^ | 2,599.69 | 259.97 | ^7,8, expert opinion^ | 503.38 | 50.34 | ^9, expert opinion^ |
| Annual cost: RAASi therapy, optimal dose ^h^ | 102.35 | 10.24 | ^13,14^ | 243.49 | 24.35 | ^13,15^ |
| Annual cost: RAASi therapy, suboptimal dose ^i^ | 52.96 | 5.30 | ^13,14^ | 144.99 | 14.50 | ^13,15^ |
| Treatment cost per day: SZC during the initiation phase | 34.96 | 3.50 | ^14,16^ | 23.26 | 2.33 | ^15,16^ |
| Treatment cost per day: SZC during the maintenance phase | 12.93 | 1.29 | ^14,16^ | 9.51 | 0.95 | ^15,16^ |
| All costs presented in Euros. SE assumed to be 10% of mean where no data available  ^a^ Four visits per year were assumed based on follow-up visits^10,17^  ^b^ 1.6 ECG, U&E test, 1.3 GP visits (38%) and 1.6 outpatient visits (14%) in Norway^7,8^ and 1.6 ECG and U&E test (52%), 1.3 GP visits (38%), and 1.6 outpatient visits (14%) in Sweden^9^, based on expert opinion  ^c^ 1.25 outpatient visits, ECG, and U&E test (75%), one visit to ER and nephrologist (75%) and 2.6 days of inpatient care (25%), with 1.5 doses of insulin and glucose (78%), one dose of calcium gluconate (40%), one dose of salbutamol and beta-adrenoreceptor agonist (13%), one dose of loop diuretic (25%), one dose of sodium bicarbonate (50%), one dose of dialysis (10%) for admitted patients in Norway^7,8^ and 1.25 outpatient visits, ECG, and U&E test (65%), one ambulance transport and emergency care (65%) and 2.6 days of inpatient care (35%), with 1.5 doses of insulin and glucose (52%), one dose of calcium gluconate (5%), one dose of loop diuretic (28%), one dose of calcium resonium (75%), one dose of dialysis (10%) for admitted patients in Sweden^9^, based on expert opinion  ^d^ Assumed equal to resource use for serum K^+^ 6.0-6.5 mmol/L, except half of admitted patients extended inpatient care to 3 days in Norway^7,8^ and assumed equal to resource use for serum K^+^ 6.0-6.5 mmol/L, except among admitted patients 1.5 doses of insulin and glucose (100%), one dose of calcium gluconate (95%), half dose of salbutamol (25%), one dose of calcium resonium (75%) in Sweden^9^, based on expert opinion  ^e^ 2.5 GP visits, blood pressure test and U&E test (20%), 2.5 outpatient visits (44%), and 2.5 days inpatient care (36%) in Norway^7,8^ and 2 GP visits, blood pressure test and U&E test (25%), 2 outpatient visits (46%), and 2.0 days inpatient care (29%) in Sweden^9^, based on expert opinion  ^f^ 2 GP visits, blood pressure test and U&E test (33%), 2 outpatient visits (37%), and 2.5 days npatient care (30%) in Norway^7,8^ and 2 GP visits, blood pressure test and U&E test (22%), 2 outpatient visits (43%), and 2.0 days inpatient care (35%) in Sweden^9^, based on expert opinion  ^g^ 2 GP visits, blood pressure test and U&E test (35%), 2 outpatient visits (36%), and 2.5 days inpatient care (29%) in Norway^7,8^ and 2 GP visits, blood pressure test and U&E test (55%), 2 outpatient visits (30%), and 2.0 days inpatient care (10%) in Sweden^9^, based on expert opinion  ^h^ Ramipril 10 mg per day (90%), candesartan cilexetil 32 mg per day (10%), and spironolactone 50 mg per day (50%) were assumed based on ESC Clinical Practice Guidelines and drug prices in the countries^13-15^  ^i^ Ramipril 6 mg per day (90%), candesartan cilexetil 10 mg per day (10%), and spironolactone 45 mg per day (50%) were assumed based on ESC Clinical Practice Guidelines, expert opinion and drug prices in the countries^13-15^  CKD: chronic kidney disease; CV: cardiovascular; ECG: electrocardiogram; ER: emergency room; ESC: European Society of Cardiology; GP: general practitioner; RAASi: renin-angiotensin-aldosterone system inhibitor; SE: standard error; SZC: sodium zirconium cyclosilicate; U&E: urea and electrolytes | | | | | | |

## RAASi discontinuation and down-titration

Parameters used to determine the proportion of patients down-titrating RAASi to a sub-maximal dose, or discontinuing either from a maximal dose or a sub-maximal dose in response to a serum potassium elevation of ≥ 5.5 mmol/L, are summarised in Table S5.

Table S5. RAASi discontinuation and down-titration

|  | **Proportion of patients discontinuing** | | **Proportion of patients down-titrating** | | **Source** |
| --- | --- | --- | --- | --- | --- |
|  | **Mean** | **SE** | **Mean** | **SE** |  |
| From maximum RAASi dose | 0.295 | 0.004 | 0.239 | 0.004 | Epstein et al.^18^ |
| From sub-maximal RAASi dose | 0.329 | 0.002 | N/A | N/A |  |
| SE: standard error; RAASi: renin-angiotensin-aldosterone system inhibitors | | | | | |

## Adverse event costs

Costs of adverse events are presented in Table S6.

Table S6. Treatment related adverse event costs per event

| Adverse events | Norway | | Sweden | |
| --- | --- | --- | --- | --- |
|  | **Mean** | **SE** | **Mean** | **SE** |
| Oedema | 693 | 69 | 171 | 17 |
| Worsening hypertension | 407 | 41 | 347 | 35 |
| Constipation | 429 | 43 | 255 | 26 |
| Diarrhoea | 429 | 43 | 255 | 26 |
| Nausea | 425 | 43 | 283 | 28 |
| Hypomagnesaemia | 1,220 | 122 | 508 | 51 |
| Anorexia | 340 | 34 | 227 | 23 |
| Hypokalaemia | 1,220 | 122 | 508 | 51 |
| Anaemia | 915 | 91 | 470 | 47 |
| Urinary tract infection | 370 | 37 | 321 | 32 |
| Cost per event estimated based on DRG tariff 2019 in the countries^10,17^ and expert opinion, multiplied with probabilities of AEs for the treatments. All costs presented in Euros unless otherwise specified. SE assumed 10% of mean.  Oedema: [3,349 NOK (802U: Poliklinisk behandling av AMD og makulaødem med lokal medikamentinjeksjon eller fotodynamisk metode) *100%] * 2 in Norway, 1,817 SEK (X54O: Ödembehandling O) * 100% in Sweden;  Worsening hypertension: [1,965 NOK (905B: Poliklinisk konsultasjon vedr hypertensjon) *100%] * 2 in Norway, 3,691 SEK (E53O: Hypertoni O) * 100% in Sweden;  Constipation / Abdominal pain / Diarrhoea / Abdominal distention: [31,794 NOK (189: Sykdom i fordøyelsesorganene ITAD >17 år u/bk) * 2% + 2,054 NOK (906O: Poliklinisk konsultasjon vedrørende andre fordøyelsessykdommer) * 70%] * 2 in Norway, 30,267 SEK (F59E: Andra sjd matsmältorg U) * 2% + 3,010 SEK (W99O: Läkarbesök andra problem O) * 70% in Sweden;  Nausea: [2,054 NOK (906O: Poliklinisk konsultasjon vedrørende andre fordøyelsessykdommer) * 100%] * 2 in Norway, 3,010 SEK (W99O: Läkarbesök andra problem O)*100% in Sweden;  Hypomagnesaemia: [52,379 NOK (296: Ernærings- og stoffskiftesykdommer ITAD >17 år m/bk) * 2% + 32,553 NOK (297: Ernærings- og stoffskiftesykdommer ITAD >17 år u/bk) * 10% + 2,277 NOK (910O: Poliklinisk konsultasjon vedr andre endokrine/ernærings-/ stoffskiftesykdommer) * 70%] * 2 in Norway, 65,417 SEK (L39E: Andra op endokr/metabol sjd U) * 2% + 13,061 SEK (L39O: Andra op endokr/metabol sjd O) * 10% + 3,975 SEK (L99O: Läkarbesök endokr/metabol sjd O) * 70% in Sweden;  Anorexia: [2,054 NOK (906O: Poliklinisk konsultasjon vedrørende andre fordøyelsessykdommer) * 80%] * 2 in Norway, 3,010 SEK (W99O: Läkarbesök andra problem O) * 80% in Sweden;  Hypokalaemia: [52,379 NOK (296: Ernærings- og stoffskiftesykdommer ITAD >17 år m/bk) * 2% + 32,553 NOK (297: Ernærings- og stoffskiftesykdommer ITAD >17 år u/bk) * 10% + 2,277 NOK (910O: Poliklinisk konsultasjon vedr andre endokrine/ernærings-/ stoffskiftesykdommer) * 70%] * 2 in Norway, 65,417 SEK (L39E: Andra op endokr/metabol sjd U) * 2% + 13,061 SEK (L39O: Andra op endokr/metabol sjd O) * 10% + 3,975 SEK (L99O: Läkarbesök endokr/metabol sjd O) * 70% in Sweden;  Anaemia: [4,421 NOK (816R: Transfusjon av fullblod eller røde blodlegemer) *100%] * 2 in Norway, 4,997 SEK (R06O: Sjd i röda blodkroppar >17 O) *100% in Sweden;  Urinary tract infection: [1,786 NOK (911O: Poliklinisk konsultasjon vedr andre sykdommer i nyre og urinveier) * 100%] * 2 in Norway, 3,407 SEK (M40O: Njur- & urinvägsinf O) * 100% in Sweden.  AE: adverse event; NOK: Norwegian krone; SE: standard error; SEK: Swedish krona; SZC: sodium zirconium cyclosilicate | | | | |

## Health-related quality of life

Health state utilities and event disutilities, and their sources, are presented in Table S7.

Table S7. Health state utilities and events disutilities per cycle (28 days)

| **Parameter** | **Mean** | **SE** | **Source** |
| --- | --- | --- | --- |
| Health state utility: CKD 3a | 0.870 | 0.034 | ^19^ |
| Health state utility: CKD 3b | 0.870 | 0.034 | ^19^ |
| Health state utility: CKD 4 | 0.850 | 0.029 | ^19^ |
| Health state utility: CKD 5 (pre-RRT) | 0.570 | 0.057 | ^20^ |
| Health state utility: Dialysis | 0.460 | 0.046 | ^20,21^ |
| Health state utility: Transplant | 0.710 | 0.019 | ^20^ |
| Event disutility: Hyperkalaemia | 0.000 | 0.000 | ^Assumption^ |
| Event disutility: Arrhythmia | -0.025 | 0.005 | ^22^ |
| Event disutility: CV event, year 1 | -0.321 | 0.032 | ^23-25^ |
| Event disutility: CV event, year 2+ | -0.192 | 0.019 | ^26^ |
| Event disutility: Hospitalisation (CKD population) | -0.024 | 0.007 | ^27^ |
| Event disutility: Dialysis complications | -0.060 | 0.006 | ^28,29^ |
| AE disutility: Worsening hypertension | -0.038 | 0.004 | ^22^ |
| AE disutility: Oedema (generalised and peripheral) | -0.038 | 0.004 | ^22^ |
| AE disutility: Constipation | -0.073 | 0.009 | ^22^ |
| AE disutility: Diarrhoea | -0.010 | 0.006 | ^30^ |
| AE disutility: Nausea | -0.048 | 0.016 | ^31^ |
| AE disutility: Hypomagnesaemia | -0.010 | 0.022 | ^22^ |
| AE disutility: Hypokalaemia | 0.000 | 0.000 | ^Assumption^ |
| AE disutility: Anaemia | -0.020 | 0.009 | ^22^ |
| AE disutility: Urinary tract infection | -0.005 | 0.007 | ^22^ |
| SE assumed to be 10% of mean where no data available.  AE: Treatment-related adverse event; CKD: chronic kidney disease, CV: cardiovascular, RRT; renal replacement therapy; SE: standard error | | | |

## Probabilistic sensitivity analysis

Table S8. Variables and distributions sampled in the PSA

| Variable | Distribution |
| --- | --- |
| Proportion female | Beta |
| Baseline age (years) | Normal |
| Baseline eGFR (mL/min/1.73 m^2^) | Normal |
| Annual probability treatment discontinuation | Beta |
| Proportion of patients with each adverse event^a^ | Beta |
| Annual cost of each adverse event^a^ | Gamma |
| Health state costs^b^ | Gamma |
| Event, AE and treatment disutilities^c^ | Beta |
| Health state utilities^c^ | Beta |
| Proportion that discontinue RAASi | Beta |
| Proportion that downtitrate RAASi | Beta |
| Proportion that uptitrate RAASi | Beta |
| Time to RAASi uptitration (weeks) | Normal |
| ^a^ modelled adverse events can be found in Table S3.  ^b^ modelled health state costs can be found in Table S4.  ^c^ modelled health state utilities and event/AE disutilities can be found in Table S7  Note that dialysis-related costs and utilities were not included in the PSA because patients in the base case exited the model before RRT initiation.  Distribution parameters calculated according to the method of Briggs et al, 2006.^32^ | |

# Supplementary results

Disaggregated results for the cost-effectiveness analysis of selected clinical scenarios are presented in Table S9 for serum K⁺ treatment threshold ≥5.1 mmol/L, Table S10 for patients initiating in the model with CKD stage 3b or stage 4, and Table S11 for the modelled scenario where patients could progress to renal replacement therapy. These results supplement those presented in Table 3.

Table S9. Disaggregated results of cost effectiveness of scenario with serum potassium treatment threshold ≥5.1 mmol/L

|  | **Norway** | | | **Sweden** | | |
| --- | --- | --- | --- | --- | --- | --- |
|  | **SZC** | **Usual care** | **Difference** | **SZC** | **Usual care** | **Difference** |
| Total costs | 79,951 | 73,864 | 6,087 | 61,988 | 57,210 | 4,777 |
| Treatment | 11,214 | 66 | 11,148 | 8,539 | 33 | 8,506 |
| Adverse events | 689 | 30 | 658 | 383 | 18 | 365 |
| Hyperkalaemia | 6,163 | 12,580 | -6,417 | 4,448 | 8,727 | -4,279 |
| CKD | 35,916 | 34,555 | 1,361 | 21,618 | 20,767 | 851 |
| RRT ^a^ | 0 | 0 | 0 | 0 | 0 | 0 |
| Arrhythmia | 1,466 | 1,401 | 64 | 1,164 | 1,112 | 52 |
| CV | 5,087 | 5,080 | 7 | 6,578 | 6,515 | 63 |
| Hospitalisation | 10,436 | 10,958 | -522 | 16,498 | 17,307 | -809 |
| RAASi use change | 8,981 | 9,193 | -212 | 2,761 | 2,733 | 28 |
| Total LYs | 4.925 | 4.728 | 0.197 | 5.056 | 4.849 | 0.206 |
| Total QALYs | 3.041 | 2.916 | 0.125 | 3.053 | 2.925 | 0.128 |
| QALYs | 4.071 | 3.910 | 0.161 | 4.103 | 3.936 | 0.166 |
| Health state (CKD) | -0.726 | -0.696 | -0.030 | -0.739 | -0.707 | -0.031 |
| Adverse events | -0.002 | -0.002 | 0.000 | -0.002 | -0.002 | 0.0000 |
| Arrhythmia | -0.001 | -0.001 | 0.000 | -0.001 | -0.001 | 0.0000 |
| CV | -0.297 | -0.291 | -0.007 | -0.304 | -0.297 | -0.0071 |
| Hospitalisation | -0.004 | -0.005 | 0.000 | -0.004 | -0.005 | 0.0002 |
| Cost/LY | 30,958 | | | 23,168 | | |
| Cost/QALY (ICER) | 48,862 | | | 37,253 | | |
| Threshold for initiating treatment for initial and recurrent hyperkalaemia events: serum K⁺ ≥ 5.1 mmol/L. All costs presented in Euros.  ^a^ In this scenario, all patients exited the model before commencing RRT.  CKD: chronic kidney disease; CV: cardiovascular; ICER: incremental cost-effectiveness ratio; LY: life year; QALY: quality-adjusted life year; RAASi: renin-angiotensin-aldosterone system inhibitor; RRT: renal replacement therapy; SZC: sodium zirconium cyclosilicate | | | | | | |

Table S10. Disaggregated results of cost effectiveness of scenario with patients initiating in the model at CKD stage 3a or stage 4

|  | **CKD stage 3a** | | | | | | **CKD stage 4** | | | | | |
| --- | --- | --- | --- | --- | --- | --- | --- | --- | --- | --- | --- | --- |
|  | **Norway** | | | **Sweden** | | | **Norway** | | | **Sweden** | | |
|  | **SZC** | **Usual care** | **Difference** | **SZC** | **Usual care** | **Difference** | **SZC** | **Usual care** | **Difference** | **SZC** | **Usual care** | **Difference** |
| Total costs | 104,546 | 101,983 | 2,563 | 82,625 | 78,926 | 3,699 | 57,045 | 55,733 | 1,312 | 42,823 | 41,634 | 1,190 |
| Treatment | 20,323 | 137 | 20,187 | 15,707 | 68 | 15,639 | 7,854 | 51 | 7,803 | 5,899 | 25 | 5,874 |
| Adverse events | 1,251 | 63 | 1,188 | 706 | 37 | 669 | 484 | 24 | 460 | 265 | 14 | 252 |
| Hyperkalaemia | 14,005 | 34,803 | -20,798 | 9,867 | 23,681 | -13,814 | 5,044 | 12,470 | -7,426 | 3,452 | 8,263 | -4,811 |
| CKD | 40,405 | 37,975 | 2,429 | 26,561 | 24,960 | 1,600 | 23,447 | 21,752 | 1,696 | 13,301 | 12,328 | 973 |
| RRT ^a^ | 0 | 0 | 0 | 0 | 0 | 0 | 0 | 0 | 0 | 0 | 0 | 0 |
| Arrhythmia | 2,169 | 2,136 | 33 | 1,746 | 1,716 | 30 | 798 | 776 | 22 | 624 | 606 | 18 |
| CV | 5,401 | 5,342 | 58 | 7,559 | 7,383 | 176 | 3,662 | 3,611 | 51 | 4,220 | 4,115 | 105 |
| Hospitalisation | 10,742 | 11,196 | -454 | 17,387 | 18,092 | -705 | 8,245 | 9,009 | -764 | 12,772 | 13,940 | -1,168 |
| RAASi use change | 10,251 | 10,330 | -80 | 3,092 | 2,989 | 103 | 7,511 | 8,041 | -530 | 2,289 | 2,343 | -53 |
| Total LYs | 7.207 | 6.821 | 0.385 | 7.500 | 7.085 | 0.415 | 2.690 | 2.496 | 0.195 | 2.720 | 2.521 | 0.199 |
| Total QALYs | 4.629 | 4.384 | 0.245 | 4.720 | 4.461 | 0.259 | 1.493 | 1.383 | 0.110 | 1.469 | 1.359 | 0.110 |
| QALYs | 5.928 | 5.616 | 0.312 | 6.068 | 5.736 | 0.332 | 2.247 | 2.087 | 0.160 | 2.220 | 2.059 | 0.161 |
| Health state (CKD) | -0.902 | -0.848 | -0.055 | -0.932 | -0.873 | -0.059 | -0.620 | -0.578 | -0.043 | -0.618 | -0.574 | -0.044 |
| Adverse events | -0.004 | -0.005 | 0.001 | -0.004 | -0.005 | 0.0006 | -0.001 | -0.002 | 0.000 | -0.001 | -0.002 | 0.0002 |
| Arrhythmia | -0.001 | -0.001 | 0.000 | -0.001 | -0.001 | 0.0000 | 0.000 | 0.000 | 0.000 | 0.000 | 0.000 | 0.0000 |
| CV | -0.386 | -0.373 | -0.013 | -0.406 | -0.391 | -0.0148 | -0.129 | -0.121 | -0.007 | -0.128 | -0.121 | -0.0074 |
| Hospitalisation | -0.005 | -0.005 | 0.000 | -0.005 | -0.005 | 0.0002 | -0.003 | -0.003 | 0.000 | -0.003 | -0.003 | 0.0003 |
| Cost/LY | 6,650 | | | 8,910 | | | 6,744 | | | 5,979 | | |
| Cost/QALY (ICER) | 10,457 | | | 14,259 | | | 11,908 | | | 10,798 | | |
| All costs presented in Euros.  ^a^ In this scenario, all patients exited the model before commencing RRT.  CKD: chronic kidney disease; CV: cardiovascular; ICER: incremental cost-effectiveness ratio; LY: life year; QALY: quality-adjusted life year; RAASi: renin-angiotensin-aldosterone system inhibitor; RRT: renal replacement therapy; SZC: sodium zirconium cyclosilicate | | | | | | | | | | | | |

Table S11. Disaggregated results of cost effectiveness of scenario with patients permitted to progress to renal replacement therapy

|  | **Norway** | | | **Sweden** | | |
| --- | --- | --- | --- | --- | --- | --- |
|  | **SZC** | **Usual care** | **Difference** | **SZC** | **Usual care** | **Difference** |
| Total costs | 138,549 | 128,573 | 9,976 | 114,940 | 105,370 | 9,570 |
| Treatment | 13,767 | 90 | 13,677 | 10,490 | 44 | 10,446 |
| Adverse events | 847 | 41 | 806 | 471 | 24 | 447 |
| Hyperkalaemia | 9,270 | 22,371 | -13,101 | 6,440 | 15,038 | -8,598 |
| CKD | 35,257 | 32,790 | 2,467 | 21,214 | 19,697 | 1,517 |
| RRT | 53,127 | 46,240 | 6,887 | 49,135 | 42,636 | 6,499 |
| Arrhythmia | 1,448 | 1,395 | 53 | 1,149 | 1,105 | 44 |
| CV | 5,063 | 4,996 | 67 | 6,524 | 6,348 | 176 |
| Hospitalisation | 10,594 | 11,255 | -660 | 16,741 | 17,753 | -1,012 |
| RAASi use change | 9,174 | 9,394 | -220 | 2,775 | 2,725 | 50 |
| Total LYs | 7.195 | 6.558 | 0.636 | 7.649 | 6.954 | 0.694 |
| Total QALYs | 4.152 | 3.798 | 0.354 | 4.322 | 3.942 | 0.380 |
| QALYs | 5.854 | 5.345 | 0.508 | 6.130 | 5.580 | 0.550 |
| Health state (CKD) | -1.401 | -1.260 | -0.141 | -1.500 | -1.345 | -0.155 |
| Adverse events | -0.003 | -0.003 | 0.000 | -0.003 | -0.003 | 0.0003 |
| Arrhythmia | -0.001 | -0.001 | 0.000 | -0.001 | -0.001 | 0.0000 |
| CV | -0.294 | -0.279 | -0.014 | -0.300 | -0.285 | -0.0154 |
| Hospitalisation | -0.004 | -0.005 | 0.000 | -0.005 | -0.005 | 0.0003 |
| Cost/LY | 15,679 | | | 13,783 | | |
| Cost/QALY (ICER) | 28,211 | | | 25,179 | | |
| Threshold for initiating treatment for initial and recurrent hyperkalaemia events: serum K⁺ ≥ 5.1 mmol/L. All costs presented in Euros.  CKD: chronic kidney disease; CV: cardiovascular; ICER: incremental cost-effectiveness ratio; LY: life year; QALY: quality-adjusted life year; RAASi: renin-angiotensin-aldosterone system inhibitor; RRT: renal replacement therapy; SZC: sodium zirconium cyclosilicate | | | | | | |

# Supplementary references

1. Kosiborod M, Rasmussen HS, Lavin P, et al. Effect of sodium zirconium cyclosilicate on potassium lowering for 28 days among outpatients with hyperkalemia: the HARMONIZE randomized clinical trial. Jama. 2014;312(21):2223-33.

2. Spinowitz BS, Fishbane S, Pergola PE, et al. Sodium Zirconium Cyclosilicate among Individuals with Hyperkalemia: A 12-Month Phase 3 Study. Clin J Am Soc Nephrol. 2019;14(6):798-809.

3. Roger SD, Spinowitz BS, Lerma EV, et al. Efficacy and Safety of Sodium Zirconium Cyclosilicate for Treatment of Hyperkalemia: An 11-Month Open-Label Extension of HARMONIZE. Am J Nephrol. 2019;50(6):473-80.

4. Nasir K, Ahmad A. Treatment of hyperkalemia in patients with chronic kidney disease: a comparison of calcium polystyrene sulphonate and sodium polystyrene sulphonate. J Ayub Med Coll Abbottabad. 2014;26(4):455-8.

5. Eriksson D, Karlsson L, Eklund O, et al. Real-world costs of autosomal dominant polycystic kidney disease in the Nordics. BMC Health Serv Res. 2017;17(1):560.

6. Södra_Regionvårdsnämnden. REGIONALA PRISER OCH ERSÄTTNINGAR FÖR SÖDRA SJUKVÅRDSREGIONEN 2019. 2018-12-06.

7. Helsedirektoratet. Innsatsstyrt finansiering (ISF) – regelverk 2018. 2018. Available from: <https://helsedirektoratet.no/finansieringsordninger/innsatsstyrtfinansiering-isf-og-drg-systemet>.

8. Den_Norsek_Legeforening. Normaltariffen. Normaltariff for fastleger og legevakt 2018-2019. 2018.

9. Södra_Regionvårdsnämnden. REGIONALA PRISER OCH ERSÄTTNINGAR FÖR SÖDRA SJUKVÅRDSREGIONEN 2018. 2017-11-30.

10. Socialstyrelsen. Prospektiva viktlistor 2019. <http://wwwsocialstyrelsense>. 2019.

11. Wisløff TS, R.M.; Halvorsen, S.: Kristiansen, I.S. Norwegian Cardiovascular Disease Model (NorCaD) – a simulation model for estimating health benefi ts and cost consequences of cardiovascular interventions. Norwegian Knowledge Centre for the Health Services (Kunnskapssenteret). 2008;No 23–2008.

12. Janzon M, Henriksson M, Hasvold P, et al. Long-term resource use patterns and healthcare costs after myocardial infarction in a clinical practice setting: results from a contemporary nationwide registry study. European Heart Journal - Quality of Care and Clinical Outcomes. 2016;2(4):291-8.

13. Ponikowski P, Voors AA, Anker SD, et al. 2016 ESC Guidelines for the diagnosis and treatment of acute and chronic heart failure: The Task Force for the diagnosis and treatment of acute and chronic heart failure of the European Society of Cardiology (ESC) Developed with the special contribution of the Heart Failure Association (HFA) of the ESC. Eur Heart J. 2016;37(27):2129-200.

14. Statens_Legemiddelverk. Legemiddelsøk. May-2019.

15. Tandvårds-_och_Läkemedelsförmånsverket. Läkemedel Databasen. May-2019.

16. Fishbane SPPP, D. K.; Roger, S. D.; Lerma, E. V.; Butler, J.; Von Haehling, S.; Spinowitz, B. S.; Adler, S. H.; Singh, B.; Lavin, P. T.; McCullough, P. A.; Kosiborod, M.;. Long-term Efficacy and Safety of Sodium Zirconium Cyclosilicate for Hyperkalemia: a 12-Month, Open-Label, Phase 3 Study. American Society of Nephrology. 2017.

17. Helsedirektoratet. Innsatsstyrt finansiering (ISF) – regelverk 2019. <https://wwwhelsedirektoratetno>. 2019.

18. Epstein M, Reaven NL, Funk SE, et al. Evaluation of the treatment gap between clinical guidelines and the utilization of renin-angiotensin-aldosterone system inhibitors. Am J Manag Care. 2015;21(11 Suppl):S212-20.

19. Gorodetskaya I, Zenios S, Mcculloch CE, et al. Health-related quality of life and estimates of utility in chronic kidney disease. Kidney international. 2005;68(6):2801-8.

20. Lee AJ, Morgan CL, Conway P, et al. Characterisation and comparison of health-related quality of life for patients with renal failure. Current medical research and opinion. 2005;21(11):1777-83.

21. SVENSKT_NJURREGISTER. SVENSKT NJURREGISTER ÅRSRAPPORT 2018. ISSN 2002-4584. 2018.

22. Sullivan PW, Slejko JF, Sculpher MJ, et al. Catalogue of EQ-5D scores for the United Kingdom. Medical Decision Making. 2011;31(6):800-4.

23. Haacke C, Althaus A, Spottke A, et al. Long-term outcome after stroke: evaluating health-related quality of life using utility measurements. Stroke. 2006;37(1):193-8.

24. Holland R, Rechel B, Stepien K, et al. Patients' self-assessed functional status in heart failure by New York Heart Association class: a prognostic predictor of hospitalizations, quality of life and death. Journal of cardiac failure. 2010;16(2):150-6.

25. Lacey EA, Musgrave RJ, Freeman JV, et al. Psychological morbidity after myocardial infarction in an area of deprivation in the UK: evaluation of a self-help package. European Journal of Cardiovascular Nursing. 2004;3(3):219-24.

26. Scotland G, Cruickshank M, Jacobsen E, et al. Multiple-frequency bioimpedance devices for fluid management in people with chronic kidney disease receiving dialysis: a systematic review and economic evaluation. National Institute for Health Research. 2018;22(1).

27. Göhler A, Geisler BP, Manne JM, et al. Utility Estimates for Decision–Analytic Modeling in Chronic Heart Failure—Health States Based on New York Heart Association Classes and Number of Rehospitalizations. Value in Health. 2009;12(1):185-7.

28. NICE. Clinical guideline [CG125]: Chronic kidney disease (stage 5): peritoneal dialysis. . 2011.

29. Sennfalt K, Magnusson M, Carlsson P. Comparison of hemodialysis and peritoneal dialysis--a cost-utility analysis. Peritoneal Dialysis International. 2002;22(1):39-47.

30. Sønbø Kristiansen I, Kristian Kvien T, Nord E. Cost effectiveness of replacing diclofenac with a fixed combination of misoprostol and diclofenac in patients with rheumatoid arthritis. Arthritis & Rheumatism. 1999;42(11):2293-302.

31. Nafees B, Stafford M, Gavriel S, et al. Health state utilities for non small cell lung cancer. Health and quality of life outcomes. 2008;6(1):84.

32. Briggs A, Claxton K, Sculpher MJ. Decision modelling for health economic evaluation. Oxford University Press. 2006.
